# NoiBene, a Group Intervention for Promoting Mental Health Among University Students: A Study Protocol for a Randomized Controlled Trial

**DOI:** 10.3389/fpsyg.2022.877340

**Published:** 2022-05-06

**Authors:** Micaela Di Consiglio, Sheila Merola, Chiara Satta, Tiziana Pascucci, Cristiano Violani, Alessandro Couyoumdjian

**Affiliations:** Department of Psychology, Faculty of Medicine and Psychology, Sapienza University of Rome, Rome, Italy

**Keywords:** university students, mental health, well-being promotion, prevention, group intervention, guided self-help intervention, help-seeking behavior, web-based intervention

## Abstract

University students’ mental health has become a public health issue since increasingly students report high levels of psychological distress. Mental health difficulties influence students’ lives, such as academic performance, relationships satisfaction, and quality of life. Moreover, different kinds of obstacles often hinder help-seeking behavior. Such evidence strongly suggests the need to implement prevention and promotion strategies to increase health and well-being in educational contexts. This article presents a study protocol for implementing and evaluating NoiBene, an evidence-based group intervention that aims to promote mental health and well-being, improve a series of transversal competencies (e.g., emotional awareness, commitment to values, assertiveness, goal setting), and decrease dysfunctional transdiagnostic mechanisms (i.e., perfectionism, repetitive thinking, experiential avoidance). A randomized controlled trial will be conducted to evaluate the protocol’s efficacy. Participants will be assigned to one of the three conditions: the NoiBene Group condition (NB-G), the NoiBene guided web-based condition (NB-WB), or the waiting list condition (WLC). The NB-G intervention consists of six face-to-face group meetings, each dedicated to specific issues related to well-being or vulnerabilities. Every meeting includes an explanation of the theoretical contents, individual and group exercises, and role-plays. The NB-WB intervention covers the same topic addressed in the NB-G intervention. In this case, participants carry out a series of online modules, including theoretical explanations, practical exercises, useful activities, and self-monitoring tools. Students will individually meet the Tutor once every 2 weeks. The primary outcome will include an increase in mental health and well-being. Secondary outcomes will include changes in emotional awareness, assertiveness, perfectionism, rumination, worry, self-criticism, experiential avoidance, and academic performance and satisfaction. We expect that participants in both NoiBene conditions will show these outcomes. However, we hypothesized that the NB-G conditions will be more effective than the NB-WB in improving assertiveness. Besides treatment efficacy, we expect that students can benefit from the NB-G or NB-WB differently based on their specific behavioral and motivational patterns. Outcomes will be assessed at pre-, post-intervention and 6-months follow-up. In conclusion, we believe that NoiBene is a promising tool that can improve students’ well-being, and it could have positive implications for preventing mental health disorders among students.

## Introduction

The last decades have been characterized by a growing interest in university students’ mental health. Several studies have amply demonstrated that psychological distress is growing among students worldwide and that a substantial number of university students meet the criteria for different mental disorders during their university years (see Sharp and Theiler, 2018 for a review). Given that most mental disorders first appear in early adulthood, particularly between 17 and 24 years (Kessler et al., 2007; De Girolamo et al., 2019), it is not surprising that data from many epidemiological studies show high levels of psychological problems among college students. Furthermore, attending university can be a stressful experience for many students that can find themselves having to cope with academic pressure and new adult-like responsibilities (Pedrelli et al., 2015; Auerbach et al., 2018; Mortier et al., 2018). High levels of psychological distress are associated with substantial impairment in academic performance (Auerbach et al., 2016; Bruffaerts et al., 2018) and represents a risk factor of dropping out of studies (Kitzrow, 2003; Ishii et al., 2018). Furthermore, mental health problems can increase vulnerability to suicidal thoughts and behaviors (Mortier et al., 2018). Despite high levels of psychological disease, many students rarely ask for help. The low rate for seeking treatment could be due to several factors that constitute the principal barriers to treatment, such as, high costs required for psychological support, stigma or self-stigma, poor mental health literacy and shortage of mental health services (Eisenberg et al., 2007, 2009). Poor help-seeking behavior is a cause of concern especially considering that untreated psychological disorders can lead to a worse outcome (Kisely et al., 2006; Ricky and O’Donnell Siobhan, 2017) and could have a long-term negative impact on a student’s quality of life.

Based on this evidence, the psychological distress that students experience can be considered a public health issue. Moreover, the recent COVID-19 outbreak impacted higher education, forcing once the closure of educational institutions and then the respect of rules such as social distancing and the use of masks (de Oliveira Araújo et al., 2020). The COVID-19 pandemic and the stringent rules to contain the virus spread had deleterious effects on students’ mental health. Indeed, many students reported an increased level of stress, anxiety, and depression during the COVID-19 pandemic (Dawson and Golijani-Moghaddam, 2020; Charbonnier et al., 2021; Di Consiglio et al., 2021b; Fernández-Castillo, 2021; Le Vigouroux et al., 2021). These data are particularly relevant if we consider the students’ mental health conditions before the pandemic (Sharp and Theiler, 2018). The increasing psychological distress showed by students during and after the pandemic reinforced still more the need to improve strategies to promote students’ mental health and prevent mental disorders. As suggested by the World Health Organization (WHO), mental health is not just the absence of mental disorders but is a state of well-being in which the individual can realize his/her-self and cope with the daily demands (World Health Organization [WHO], 2018). From this perspective, mental health promotion and prevention are necessarily related and overlapping: mental health is promoted by increasing well-being and enhancing transversal competencies, and mental disorders are prevented by reducing risk factors (Lahtinen et al., 1999; Saxena et al., 2006). University is an essential part of any health promotion and prevention strategy: it can develop a culture that supports mental health, it can provide students with mental health service, and it can build an environment that facilitates help-seeking behavior (Tsouros et al., 1998; Hughes and Spanner, 2019). For example, to promote well-being in the educational context, the World Health Organization [WHO] (1994) has focused on developing and/or strengthening a series of cognitive, emotional, and relational transversal skills, also called life skills. Life skills include making decisions and solving problems, critical and creative thinking, communication and interpersonal relationships, self-awareness and empathy, and emotion regulation. A broad range of evidence suggested the effectiveness of life skills training among university students in reducing the impact of stressful events, in increasing mental health (Savoji and Ganji, 2013), emotional intelligence (Lolaty et al., 2012), happiness, quality of life and emotion regulation (Mohammadkhani and Hahtami, 2011), in preventing drugs abuse behaviors (Moshki et al., 2014), in decreasing anxiety and depression symptoms (Sobhi-Gharamaleki and Rajabi, 2010).

In line with these considerations, the last decades have been characterized by a growing spread of promoting and preventing intervention among university students (Conley et al., 2013; Cuijpers et al., 2021). For example, but not limited to, such interventions are effective in increasing positive mental health and decreasing depression and anxiety symptoms (Bendtsen et al., 2020), in reducing distress (Galante et al., 2018), in promoting self-compassion, social connectedness, resilience, and flourishing (Long et al., 2021) and in enhancing self-acceptance, positive relations with others, optimism, and self-esteem (Marrero et al., 2016). Most of these interventions have been developed to overcome the costs and the limits of the traditional form of intervention, such as one-to-one in-person treatment, provided in a clinical setting and administered by a highly trained mental health professional (Kazdin, 2019). More specifically, these models of treatment, generally developed as internet-based intervention or group intervention, can reach a more significant number of people, and could represent an effective solution to address the issue of mental health among students. Both internet-based intervention and group intervention have a series of advantages: internet-based intervention, generally delivered *via* computer or smartphone, allows access to a series of resources at any time and from all places, speedily and easily; it guarantees anonymity, privacy, and confidentiality and it is a low-priced tool (Barak and Grohol, 2011; Montagni et al., 2020). Moreover, internet-based intervention is particularly leading among young people, especially students, since they represent one of the largest consumers of digital devices (Lenhart et al., 2010). As well as that previously described, group interventions are generally cost-effective; consequently, they can increase the accessibility to services and reduce barriers to treatment. Moreover, unlike internet-based interventions, they have the advantage of allowing students to interact with others and to promote relationships between group members working together toward common goals. Some factors, such as socializing techniques and interpersonal learning that are crucial elements of group interventions (Yalom, 1985), may be essential for university students to improve social skills to develop more positive and effective relationships, both in personal and professional contexts. Several studies have shown that group interventions in college students are effective for the treatment of depression (Church et al., 2012), for the development of resilience and coping techniques (Houston et al., 2017), for facilitating the transition of students to the first year of university (Oppenheimer, 1984), to promote social support, self-efficacy, understanding of one’s own and other’s emotions (Ando, 2011). As exposed, both modalities have a series of advantages, however, although to some extent effective, most of these programs focus on specific issues or disorders, thus underestimating the relevance of comorbidity among mental disorders (Kessler et al., 2005; Roca et al., 2009; Nock et al., 2010), between mental and medical conditions (Alegria et al., 2001; Daré et al., 2019; Kelly et al., 2019) and the interaction among risk factors, protective factors, and stressful events (Belsky and Pluess, 2009). Moreover, such interventions foster a mechanistic view of the understanding of the human being, which contrasts with the biopsychosocial model of health and the development of personalized interventions (Frazier, 2020). For these reasons, a change of strategy appears necessary. Such interventions should aim to globally promote health and psychological well-being, in terms of individual’s resources, abilities and strengths, together with a transdiagnostic approach (Mansell et al., 2009): it can be helpful in early intercepting psychopathological trajectories and prevent the risk of developing mental disorders and psychological distress. Based on these considerations, we developed NoiBene, a comprehensive and wide-ranging intervention that aims to promote well-being and prevent various forms of psychological distress by targeting some of the main risk and maintaining factors for mental disorders (Di Consiglio et al., 2021a). NoiBene has been developed on evidence-based approaches such as Positive Psychology (Seligman et al., 2005), Cognitive–Behavioral Therapies (CBT; Dobson and Dobson, 2018), and Nonviolent Communication (NVC; Rosenberg and Chopra, 2015). Positive psychology promotes adaptive human functioning by fostering positive emotions, behaviors, and character strengths that allow individuals to build a life of meaning and purpose (Seligman et al., 2005). CBTs, especially Acceptance and Commitment Therapy (Hayes et al., 2009) and Self-Compassion Therapy (Gilbert, 2009), are expanding their target from the mere reduction of symptomatology to the development of skills that contribute to mental health and well-being, such as self-acceptance and commitment to personal values. Lastly, NVC is a method based on judgment-free observation and recognition of feelings and needs that aim to promote empathic communication fostering positive interpersonal relationships (Rosenberg and Chopra, 2015). NoiBene integrates these theoretical and intervention models: the aim is to operate according to a promotion and prevention approach. Especially, the program aims (1) to improve mental health and well-being, (2) to increase positive and flexible behaviors, (3) to improve those life skills mainly related to psychological well-being and self-realization (e.g., emotional awareness, assertive communication, goal setting), (4) to promote the commitment to the satisfaction of personal needs and values, (5) to enhance strategies to adequately handle academic challenges, (6) to weaken some of the main risk and maintaining factors for mental disorders (i.e., repetitive thinking, perfectionism and experiential avoidance).

### Aim and Scope

The purpose of this paper is to present the research protocol and recruitment process that will be employed for a randomized controlled trial (RCT) to evaluate the efficacy of NoiBene as a group intervention (NB-G). This condition will be compared with two other conditions: NoiBene as a guided web-based intervention (NB-WB) and with a waiting list condition (WLC). We hypothesized that participants in the NoiBene conditions (both NB-G and NB-WB), compared with participants in the WLC, would show after the intervention higher levels of mental health and well-being (Cuijpers et al., 2021) as well as an increase in academic performance and satisfaction (Bean and Eaton, 2001). We also predict that students in the NoiBene conditions will increase their emotional awareness and assertiveness, and they will reduce the level of perfectionism, repetitive thinking, and experiential avoidance (Di Consiglio et al., 2021a). However, we hypothesized that the NB-G conditions will be more effective than the NB-WB in improving assertiveness, since the group context guarantees more supervised practice (Salas and Cannon-Bowers, 2001; Gresham, 2002; Taylor et al., 2005), and it ensures a positive environment where the member can interact in an adaptive manner (Yalom, 1985). However, besides treatment efficacy, we expect that students can benefit from the NB-G or NB-WB differently based on their specific behavioral and motivational patterns. For example, bases on specific personality traits or set of values students could be more motivated to participate and benefit more from a guided web-based intervention or from a group intervention.

## Methods

### Participants

Seventy-five (*n* = 75) students will be voluntarily recruited from different universities in Rome, Italy. The sample size for this study was determined based on power analysis using G*Power 3.1.9.7 (Faul et al., 2007). We calculated that the minimum required sample size to detect an effect size of *f* = 0.25 using a mixed analysis of variance (ANOVA) including within-subject (3-time points) and between-subject (3 groups) effects with 95% power and at a two-sided 5% significance level was 54 participants (18 per treatment arm). However, attrition is a common problem that occurs in RCT: around 20% of participants withdraw before completing the study protocol (Mason, 1999). In conclusion, we plan to recruit approximately 75 participants (25 per treatment arm).

NoiBene will be advertised by word of mouth, mailing lists, and sharing fliers on various social networks (i.e., Facebook, Instagram). Students will be informed about the implementation of a group intervention to promote well-being and to prevent psychological distress. They will receive information about the program and about the specific goal of the intervention. The organization and the calendar of the meetings will also be presented. Since many university courses include credits achievable by practical activities (highly specialized workshops), all participants will receive an intervention completion certificate that they can use to achieve such credits.

### Eligibility Criteria

Participants will be eligible for participation if they will be enrolled on any degree course at the beginning of the study, be able to write, read and speak Italian, and have regular access to the internet. The exclusion criteria will be the presence of ongoing severe mental disorders according to the Diagnostic and Statistical Manual of Mental Disorders (DSM–5 - American Psychiatric Association, 2013), such as major depression, bipolar disorder, psychotic disorders. Exclusion criteria will also include the presence of suicidal ideation. To evaluate the presence of ongoing clinical disorders a series of standardized questionnaire will be use. If the questionnaire suggests elevated levels of symptomatology the student will be contacted for a diagnostic interview. If severe ongoing clinical conditions (e.g., mood disorders, psychotic disorders) or suicidal ideation will be confirmed, the student will receive feedback about his symptomatology and will be directed toward an intervention that fits more with his or her needs (i.e., psychological counseling, psychotherapy). In this case, the NoiBene intervention can be used as a support for their therapy.

### Measures

#### Measures for the Assessment

To exclude the presence of any ongoing severe mental disorders, the Symptom Checklist-90-Revised (SCL-90-R; Derogatis, 1975; Italian validation by Sarno et al., 2011), the Millon Clinical Multiaxial Inventory-III (MCMI-III; Millon, 1997; Italian validation by Zennaro et al., 2008) and the Structured Clinical Interview for DSM-5 (SCID-5-CV; First et al., 2016; Italian validation by First et al., 2017) will be used to detect mental health concerns. The SCL-90-R is a self-report questionnaire used to evaluate a broad range of symptoms of psychopathology (i.e., somatization, obsessive-compulsive, interpersonal sensitivity, depression, anxiety, hostility, phobic anxiety, paranoid ideation, and psychoticism). It also evaluates an index of overall psychological distress, an index of the intensity of symptoms, and the number of self-reported symptoms. Considering the Italian version, Cronbach’s alpha ranges from 0.68 to 0.87. The MCMI-III is a self-report questionnaire that provides information about personality disorders and clinical syndromes according to the DSM-IV-TR (American Psychiatric Association, 2000). The internal consistency of the questionnaire is moderate, with Cronbach’s alpha ranging from 0.66 to 0.95. The SCID-5-CV is a semi structured interview to make major DSM-5 diagnoses (American Psychiatric Association, 2013). The percentage of positive agreement between the interview and clinical diagnoses ranges between 73 and 97% (Osório et al., 2019).

#### Primary Outcome

Primary outcomes will include changes in psychological and mental well-being evaluated with the Psychological General Well-Being Index (PGWBI - Dupuy, 1984; Italian validation by Grossi et al., 2002) and the Warwick-Edinburgh Mental Well-being Scales (WEMWBS - Tennant et al., 2007; Italian validation by Gremigni and Stewart-Brown, 2011). The PGWBI is a 22-item Health-Related Quality of Life questionnaire that evaluates self-perceived health and psychological well-being. Especially, it evaluates six dimensions: anxiety, depressed mood, positive well-being, self-control, general health and vitality. The questionnaire provides a score for each dimension and a total score, with higher scores indicating greater psychological well-being. The questionnaire demonstrated a good internal consistency with Cronbach’s alphas ranging from 0.90 to 0.94. The WEMWBS is a 14-item scale that evaluates mental well-being, covering subjective well-being and psychological functioning. The questionnaire provides a total score reflecting aspects of positive mental health. The Italian questionnaire has a good internal consistency coefficient with a Cronbach’s alpha of 0.86.

#### Secondary Outcomes

Secondary outcomes include changes in emotional awareness, assertiveness, repetitive thinking, perfectionism, and experiential avoidance.

##### Emotional Awareness

Emotional awareness is the ability to identify, recognize, manage, and communicate one’s and others’ emotions (Lane and Schwartz, 1987). We will assess the ability to identify, recognize and communicate emotions with the Toronto Alexithymia Scale-20 (TAS-20; Bagby et al., 1994a,b; Italian validation by Bressi et al., 1996). The TAS-20 is one of the most used measures of alexithymia and it focuses on the ability to identify and describe feelings. Indeed, the questionnaire has 3 subscales: difficulty describing feelings, difficulty identifying feelings, and externally oriented thinking. It also provides a total score for alexithymia. The Italian version demonstrated good internal consistency for the total score (α = 0.81).

##### Assertiveness

To evaluate the level of assertiveness, we will use the Scale for Interpersonal Behavior (SIB; Arrindell and Van der Ende, 1985; Italian validation by Arrindell et al., 1999), a self-report questionnaire used to measure distress in state assertiveness (distress scale), and the probability of engaging in a specific assertive behavior (performance scale). The SIB contains four dimensions for each scale: negative assertion, personal limitation, initiating assertiveness, and positive assertion. It also provides an overall measure of assertion for each scale. The Italian version has internal consistency coefficients ranging from 0.73 to 0.91.

##### Repetitive Thinking

Repetitive thinking is defined as a process of passive thinking, uncontrollable, and focused on negative content. Two forms of repetitive thinking have been mostly recognized: rumination and worry (Ehring and Watkins, 2008). To assess rumination, we will use the Ruminative Response Scale (RRS; Nolen-Hoeksma and Morrow, 1991; Italian validation by Palmieri et al., 2007), one of the most used self-reported questionnaires to measure depressive rumination. To assess worry, we will use the Penn State Worry Questionnaire (PSWQ; Meyer et al., 1990; Italian validation by Morani et al., 1999), a self-administered questionnaire that aims to measure worry regarding its occurrence, pervasiveness, and intrusiveness. The Cronbach’s alpha of the Italian version was 0.85.

##### Self-Criticism

To assess self-criticism, we will use the Level of Self-Criticism (LOSC; Thompson and Zuroff, 2004; Italian validation by Manfredi et al., 2016), a self-reported questionnaire designed to measure two dysfunctional forms of self-criticism: comparative self-criticism and internalized self-criticism. The authors reported Cronbach’s alpha coefficients of 0.81 for CSC and of 0.87 for ISC.

##### Experiential Avoidance

To assess experiential avoidance, we will use the Acceptance and Action Questionnaire-II (AAQ-II; Bond et al., 2011; Italian validation by Pennato et al., 2013), one of the most used measures of psychological inflexibility. Psychological inflexibility is defined *“the rigid dominance of psychological reactions over chosen values and contingencies in guiding action”* (Bond et al., 2011, p. 678). According to the ACT model, psychological inflexibility is the cornerstone of experiential avoidance (Pennato et al., 2013). It is a 10-item questionnaire that aims to measure psychological inflexibility, a measure of experiential avoidance. The Italian version showed good internal consistency (α = 0.83).

##### Perfectionism

Perfectionism is defined as the need to reach high personal standards, accompanied by critical self-evaluations and concerns regarding others’ evaluations and mistakes (Frost et al., 1990). We will assess perfectionism using the Multidimensional Perfectionism Scale (MPS; Frost et al., 1990; Italian validation by Lombardo, 2008), a self-reported questionnaire widely used in research and clinical settings to evaluate perfectionism. The Italian version of the questionnaire includes four scales: (1) high personal standards; (2) concern over mistakes and doubt about mistakes; (3) parental expectations and parental criticism; (4) appreciation for order and organization. Cronbach’s alphas for the four scales have been reported ranging from 0.76 to 0.87.

##### Academic Performance and Satisfaction

An *ad hoc* questionnaire will be administered to evaluate academic performance (i.e., number of taken and passed exams, mean grades, percentage of attended lessions). The questionnaire will also include self-reported information about academic satisfaction (i.e., “*I feel satisfy of my academic performance”*), perceived self-efficacy (i.e., “*I can reach my academic goals,”* “*I have a good study method,”* “*I can organize my time to study”*), and to investigate concerns about the university (i.e., “*I feel anxious during the study,”* “*I feel anxious during the exams,”* “*I can not sleep the night before the exams,”* “*I spend a lot of time worrying about university,”* “*I feel anxious to speak with my colleagues,”* “*I feel anxious to speak with my professors”).*

#### Sociodemographic Questionnaire

An *ad hoc* questionnaire will be administered to collect information about the residence, professional status, psychological past/current treatment and educational level.

#### Feedback About the Program

An *ad hoc* questionnaire will be administered to collect feedback from participants in the NB-G condition after every meeting. The questionnaire consisted of 13 items, with 10 items scored on a five-point Likert scale ranging from 1 (strongly disagree/very poor) to 5 (strongly agree/excellent), and three open answer questions. The questionnaire aims to evaluate the level of satisfaction with the contents and activities proposed during the meeting, to collect opinions about the quality of the education and the ability of the Tutor to be understandable and engaging. Lastly, the questionnaire asks for any opinions and suggestions. Only at the end of the last meeting eight additional questions are presented in the questionnaire. These questions aim to investigate the perceived utility of NoiBene and collect opinions and suggestions for future developments. See Supplementary Appendix 1 for the entire questionnaire.

At the end of the program, an *ad hoc* questionnaire will be administered to collect feedback among participants in the NB-WB condition. The questionnaire consisted of 70 questions, with 63 items scored on a five-point Likert scale ranging from 1 (strongly disagree) to 5 (strongly agree), and seven open answer questions. The questionnaire aims to investigate the intervention’s perceived utility, evaluate the level of satisfaction with the contents and activities proposed, collect opinions about the Tutor, and ask for any suggestions for future developments. See Supplementary Appendix 2 for the entire questionnaire.

#### Therapeutic and Group Relationship

To evaluate therapeutic and group relationship participants in the guided web-based and group conditions will answer an adaptation of Working Alliance Inventory for guided Internet interventions (WAI-I; Gómez Penedo et al., 2020) and the Group Questionnaire (GQ; Krogel et al., 2013; Burlingame et al., 2017; Italian validation by Giannone et al., 2020), respectively.

The WAI-I is a 12-items questionnaire scored on a 5-point Likert scale. It is used for the assessment of the therapeutic alliance in the context of guided internet-based intervention. The questionnaire provides two subscales: (1) goal and task (i.e., agreement on the goal and on the task); (2) bond (i.e., development of an affective bond). The items of the WAI-I were derived from the WAI-SR (Hatcher and Gillaspy, 2006). In the adaptation the item remained similar to the original scale, but the word “session” and “therapy” were rephrased with the word “online program,” and the word “therapist” was rephrased with the word “tutor.” The Cronbach’s α for the WAI-I total score was 0.93. Regarding the two subscales, the Cronbach’s αs were 0.93 for the task and goal agreement with program dimension and 0.89 for the bond with therapist dimension. The GQ is a 30-items questionnaire scored on a 7-point Likert scale. It provides information on the quality of the relationships in the group considering three main dimensions: (1) positive bonding (i.e., sense of belonging that the member has to the group, its members, and its leader); (2) positive working (i.e., ability of the group to agree upon treatment goals); (3) negative relationship (i.e., lack of trust and understanding). It also provides specific information about the group structure since it investigates relationships in the group (member - member, member - leader, and member - group). The original questionnaire shows a good internal consistency, with Cronbach’s alpha ranging to 0.80 to 0.92 for the three dimensions.

### Procedure

**FIGURE 1:**
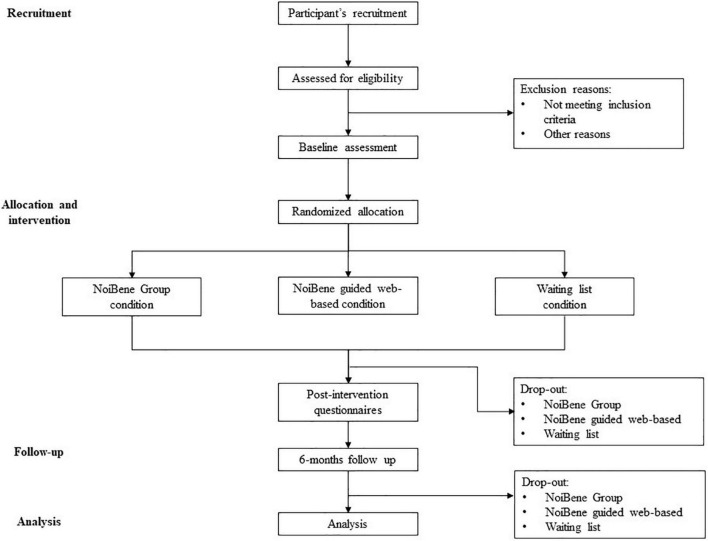
RCT flow diagram.

This study was approved by the Ethics Committee of the Department of Psychology, Faculty of Medicine and Psychology, Sapienza University of Rome (code n. 0001443). Students who desire to participate in the NoiBene have to send an email to ask for participation. Before starting the study, participants will have to provide informed consent after reading about the goals and procedure of the study and information about data protection and privacy according to the General Data Protection Regulation (EU) 2016/679 (GDPR). Participation will be voluntary, and every participant will be free to withdraw at any time. Once achieved the consent, to detect clinical disorders, students will ask to answer an online survey containing MCMI-III and SCL-90-R questionnaires. According with the exclusion criteria, if no significant clinical condition is suggested, participants will be randomly placed into one of the three conditions: the NoiBene group intervention (NB-G), the NoiBene guided web-based intervention (NB-WB), and a waiting list condition (WLC). Before and after the intervention, a series of standardized questionnaires will be administered to participants of both groups to assess the mental health and well-being (PWBI and WEMWBS), emotional awareness (TAS-20), assertive communication (SIB), perfectionism (MPS), rumination (RRS), worry (PSWQ), self-criticism (LOSC), and experiential avoidance (AAQ-II). Moreover, an *ad hoc* questionnaire will be administered to evaluate academic performance and satisfaction with the program. The same assessment will be repeated 6 months after the end of the intervention. Moreover, since therapeutic and group relationships are a powerful non-specific element that predicts outcome in individual (Lambert and Barley, 2001) and group interventions (Johnson et al., 2005), to monitor the therapeutic and group relationship, the WAI-I and the GQ will be administered to participants in the NB-WB and NB-G conditions, respectively. Figure 1 shows the recruitment and study procedure.

#### The NoiBene Group Condition

Students in this condition will receive the NoiBene Group intervention. It consists of six in-person group meetings (4 h per meeting). The meetings will be performed once every 2 weeks, for 3 months. Every meeting focus on a specific issue: (1) emotions, needs, and values; (2) repetitive thinking (worry, rumination, and self-criticism); (3) perfectionism and experiential avoidance; (4) social competence: active listening, communicative style; (5) social competence: assertiveness; (6) healthy lifestyle, goal setting, planning, and study method. Every meeting includes an explanation of the theoretical contents, individual and group exercises, and role-play. See Supplementary Table 1 in the Supplementary Material for more information about the sessions. At the end of every meeting, participants will receive a questionnaire to evaluate the level of satisfaction and to collect feedback to improve the intervention. After every meeting, students can access the website platform of NoiBene, where they can find a series of online modules regarding the same topic discussed in the meetings group with practical exercises and self-monitoring tools. Every participant will ask to complete the module regarding the topic discussed in the group meeting. The aim is to stimulate students to practice more with the exercise and techniques proposed during the meeting. Every group will be composed of a maximum of 15 students and a minimum of 10 students. The group size depends on whether the optimum number for group therapy is 8–12 members (Ezhumalai et al., 2018). We decided to include more students because we expect some degree of drop-out. The meetings will be co-delivered by two qualified psychologists. All the psychologists included in the intervention will have previous training in delivering the treatment. Moreover, considering that treatment fidelity can affect outcomes (Couturier et al., 2021), all the psychologists included in the trial will be supervised by an expert psychotherapist (the author of the treatment) to ensure treatment fidelity.

#### NoiBene Guided Web-Based Condition

Students in this condition will receive a username and password to sign up on the NoiBene website. NoiBene provides nine training modules, and each module is dedicated to a specific issue, as the same as the NB-G condition. Each module contains theoretical explanations and animated videos to engage users and facilitate learning. Moreover, students find practical exercises, useful activities and self-monitoring tools to practice the previously introduced principles. See Supplementary Table 2 in the Supplementary Material for more information about the modules (the entire protocol has been published in a previous work of Di Consiglio et al., 2021a). Students will meet the Tutor once every two weeks; the meetings will be held on video-call platforms, guaranteeing a private space. The Tutor aims: (1) to help the students carry out the NoiBene program on a constant and continuous basis to reduce the rate of drop out; (2) to maintain and stimulate the motivation and commitment of the students in carrying out the program and in the achievement of the set goal; (3) to monitor the progress of the student through the website; (4) to review the activities, to explain unclear concepts and to address any difficulties encountered. All the Tutors included in the intervention will have previous training in delivering the treatment. Even in this case, all the Tutors included in the trial will be supervised by an expert psychotherapist (who is also the author of the treatment) to ensure treatment fidelity (Couturier et al., 2021).

#### Waiting List Condition

Students in this condition will not receive any intervention. After the study will be ended, the waiting list participants will be invited to take part in the intervention. Participants randomized to the WLC will be offered to choose their preferred treatment after completing follow-up measurements. However, the beginning of the group intervention depends on whether the minimum number of participants is reached. So, we will inform students that if we do not receive at least 10–12 requests of participation, they will have only the opportunity to be included in the guided web-based intervention.

## Statistical Analysis

Data will be processed using SPSS (IBM) version 27. Descriptive analysis will be run for all the sociodemographic and academic variables, on data related to the satisfaction questionnaires and on SCL-90-R and MCMI-III scores. Chi-Square tests will be used to investigate differences between participants in the three conditions on baseline demographic variables and gender variables. With the same aim, a series of Student’s *t*-test will be run on age variable and on all baseline measures. If the test will provide significant *p*-values, effect sizes will be calculated considering Cohen’s d (small > 0.20, medium > 0.50, large > 0.80). If some variables will result significantly different between conditions, they will be included as covariant in the further analysis. A series of mixed models of analysis of variance (ANOVAs) will be run to investigate the efficacy of the intervention on the PWBI, WEMWBS, TAS-20, SIB, MPS, LOSC, RRS, PSWQ, and AAQ scores, as a function of the factor between CONDITION (NB-G vs. NB-WB vs. WLC) and the factor within TIME (Baseline vs. Post-Intervention vs. Follow-Up). The level of significance was set at *p* < 0.05. Effect sizes will be calculated using partial eta squared and it will be interpreted based on benchmarks suggested by Cohen (2013): *n*^2^ = 0.01, small effect size; *n*^2^ = 0.06, medium effect size; *n*^2^ = 0.14 large effect size. The *post hoc* analyses of significant interaction will be conducted using the Bonferroni *post hoc* test.

## Expected Results and Discussion

Mostly, mental health disorders have the first onset by 24 years (Kessler et al., 2007; De Girolamo et al., 2019). University years, which coincide with this critical period, represent a crucial moment in every student’s life. Recent evidence suggested that mental health problems are prevalent among university students (Auerbach et al., 2018). Such problems may affect students’ lives and lead to a personal, relationship, and academic difficulties (Alonso et al., 2018; Bruffaerts et al., 2018). Over the past decades, the awareness about the importance of implementing strategies for promotion and prevention has exponentially grown. In this field, we developed the NoiBene intervention, a program to improve students mental health and well-being, and to prevent mental health disorders.

We expect that participants in the NoiBene conditions will show a higher level of mental and psychological well-being after the intervention than the waiting list condition. Indeed, NoiBene includes a series of interventions to increase some fundamental life skills, such as emotional awareness, assertiveness, and goal setting, elements that have an important role in determining mental health (Savoji and Ganji, 2013). Moreover, it provides a series of interventions focusing on self-acceptance-related emotions, an important factor that positively affects mental health (Rash et al., 2011; Weiss et al., 2016). Lastly, the NoiBene conditions include a psychoeducation about healthy lifestyles; students are then encouraged to link them with their own values and to set goals according to them. A broad range of evidence suggests that healthy lifestyle interventions are associated with mental health and well-being improvements (Dale et al., 2014). We also predicted that participants in the NoiBene condition will improve their academic performance and satisfaction. Indeed, the NoiBene program includes contents about the study method, and it could contribute to improving their performance and study skills (Meneghetti et al., 2016). Moreover, the psychological well-being of university students is an important factor in successfully dealing with the demands of academic life. High levels of well-being promote the adoption of more adaptive coping strategies, such as positive reappraisal of the problems and help-seeking behaviors (Freire et al., 2016). Furthermore, higher levels of well-being positively impact personal growth and academic achievements (Bowman, 2010; Bordbar et al., 2011; Yu et al., 2018). We hypothesized that participants in the NoiBene conditions will decrease levels of repetitive thinking, perfectionism, and experiential avoidance (Di Consiglio et al., 2021a). We expect that the reduction of these dysfunctional process can reduce the level of specific symptoms such as anxiety and depression and can buffer against the development of future mental disorders (Frank and Davidson, 2014).

Moreover, we believe that the group protocol could be even more effective than the guided web-based protocol in improving assertiveness (Conley et al., 2013), since the group protocol ensures supervised skills practice over multiple sessions, an essential component of successful training (Salas and Cannon-Bowers, 2001; Gresham, 2002; Taylor et al., 2005). Moreover, some unique characteristics of the group can foster this positive change. For example, the group ensure a positive environment where the member can interact in an adaptive manner and can engage in positive relationship; also, the group context ensure the modeling process both from the Tutor and from other member and imitative behaviors are an important source of learning (Yalom, 1985; Johnson et al., 2005) unlike the individual guided intervention. However, we believe that it is important to consider that students can benefit from the NB-G or NB-WB differently based on their specific behavioral and motivational patterns. For example, values are an important broad motivator of actions and affect prosocial behavior (Schwartz, 1992). Values such as benevolence and conformity promote cooperative and supportive social relations. Indeed, these values motivate actions to promote the welfare of others and to avoid adverse outcomes for self. Conversely, values such as security and power usually oppose prosocial behavior since they motivate to maintain a stable and protective environment and emphasize self-interest (Schwartz, 2010). Students guided by such values could be more motivated to participate and benefit more from a group intervention or from a guided self-help intervention, respectively. This consideration is significant if we also consider the clinical utility of these interventions. This dimension concerns the extent to which the intervention will be effective in a real-world practical setting (American Psychological Association, 1995). Moreover, when proposing an intervention, it is important to consider not only the best available intervention but also the individual characteristics and preferences (American Psychological Association Presidential Task Force on Evidence-Based Practice, 2006). Therefore, outside a controlled empirical context, we expect that students will report a preference toward a group or guided self-help intervention based on personal characteristics.

Students in the NoiBene conditions will receive a series of theoretical information about well-being and factors that contribute to mental health or mental disorders. We expect that this increasing knowledge would foster a better attitude toward help-seeking behavior (Jorm et al., 1997; Gulliver et al., 2010). Moreover, we will conduct psychodiagnostics interviews and feedback meetings with students reporting high levels of psychological distress. In the case of psychopathology, we will have the opportunity to give personalized feedback about students’ symptomatology, discuss their personal barriers that hinder the help-seeking behavior, and inform them about how and where to find help in the area. It could be an efficacious strategy to encourage the request for help (Gulliver et al., 2010; Di Consiglio et al., 2021b).

In conclusion, NoiBene presents a series of strengths and practical implications. Although interventions targeting specific areas of functioning are important, NoiBene has the advantage that it is a comprehensive program built on a eudemonic (Ryff and Keyes, 1995) and hedonic (Kahneman et al., 1999) approach to well-being. Indeed, NoiBene aims to promote self-realization and foster positive affect. Moreover, NoiBene focuses on a series of essential transversal competence (e.g., emotional awareness, assertiveness, goal setting), covering a range of variables necessary for mental health. Second, most of the preventing trials are focused on specific issues such as depression, anxiety, and stress (see Cuijpers et al., 2021 for a review). NoiBene has been developed on a transdiagnostic approach: it considers three of the main factors occurring in many mental health disorders such as repetitive thinking, perfectionism, and experiential avoidance (Frank and Davidson, 2014). It means that the activities that NoiBene proposes do not focus on specific symptoms or disorders but instead focus on dysfunctional processes that may contribute to the development of psychopathology. For this reason, we expect that it can be helpful the reduce the risk of the development of different mental disorders. Lastly, it is important to consider that besides negative consequences on individual students’ quality of life, the high prevalence of mental disorders and psychological distress among students is also critical for universities. Indeed, it can hurt academic performance and participation (Eisenberg et al., 2007; Reavley and Jorm, 2010; Buchanan, 2012), and protracted psychological distress leads to reduced self-efficacy, motivation, and academic satisfaction (Lipson and Eisenberg, 2018). Moreover, emotional problems seem to be the most common reason students drop out during the first year of university (Baik et al., 2015). Therefore, NoiBene could provide the opportunity to integrate health into university culture and foster a positive environment that can implicate benefits for students and the entire university community.

## Author Contributions

MDC and AC: conceptualization. MDC, SM, CS, TP, CV, and AC: methodology and writing—review and editing. AC: supervision. MDC, SM, CS, and AC: writing—original draft. All authors have read and agreed to the published version of the manuscript.

## Conflict of Interest

MDC, SM, CS, and AC are members of the NoiBene Team and contributed to the development of NoiBene. AC is the scientific director of NoiBene. The remaining authors declare that the research was conducted in the absence of any commercial or financial relationships that could be construed as a potential conflict of interest.

## Publisher’s Note

All claims expressed in this article are solely those of the authors and do not necessarily represent those of their affiliated organizations, or those of the publisher, the editors and the reviewers. Any product that may be evaluated in this article, or claim that may be made by its manufacturer, is not guaranteed or endorsed by the publisher.
