# Supplementary material for: NoiBene, a Group Intervention for Promoting Mental Health Among University Students: A Study Protocol for a Randomized Controlled Trial
Source: Front Psychol. 2022 May 6;13:877340. doi: 10.3389/fpsyg.2022.877340 (PMC9120589; doi:10.3389/fpsyg.2022.877340)
Supplement: Supplementary file 1 [file Data_Sheet_1.docx]

Supplementary Material

# Appendix-1. Feedback questionnaire (NB-G condition)

| We ask you to answer the following questions concerning the meeting you just participated in. Please remember that the questionnaire is completely anonymous. Your opinion is essential to improve the program.  Please consider the following scale when answering the questions.   \| 1  Strongly disagree \| 2  Disagree \| 3  Neither agree or disagree \| 4  Agree \| 5  Strongly agree \| \| --- \| --- \| --- \| --- \| --- \| | | | | | |
| --- | --- | --- | --- | --- | --- | --- | --- | --- | --- | --- |
| I appreciate this meeting | 1 | 2 | 3 | 4 | 5 |
| I learnt from this meeting | 1 | 2 | 3 | 4 | 5 |
| The content discussed where in line with my need | 1 | 2 | 3 | 4 | 5 |
| This meeting was interactive | 1 | 2 | 3 | 4 | 5 |
| This meeting was boring (R) | 1 | 2 | 3 | 4 | 5 |
| Please consider the following scale when answering the questions.   \| 1  Very poor \| 2  Poor \| 3  Fair \| 4  Good \| 5  Excellent \| \| --- \| --- \| --- \| --- \| --- \| | | | | | |
| How did you consider the educational quality of the meeting? | 1 | 2 | 3 | 4 | 5 |
| How did you evaluate the organization of the meeting? | 1 | 2 | 3 | 4 | 5 |
| How did you consider the time dedication to discussion? | 1 | 2 | 3 | 4 | 5 |
| How did you consider the Tutor ability to engage your interest? | 1 | 2 | 3 | 4 | 5 |
| How did you consider the Tutor ability to support you during the activities? | 1 | 2 | 3 | 4 | 5 |
| Now please fill out the following open questions | | | | | |
| What did you not appreciate? | | | | | |
| What made you feel bad/embarrassed? | | | | | |
| Feedback and suggestions | | | | | |

| Additional questions for the last meeting.  Please consider the following scale when answering the questions.   \| 1  Strongly disagree \| 2  Disagree \| 3  Neither agree or disagree \| 4  Agree \| 5  Strongly agree \| \| --- \| --- \| --- \| --- \| --- \| | | | | | |
| --- | --- | --- | --- | --- | --- | --- | --- | --- | --- | --- |
| NoiBene helped me to improve my well-being | 1 | 2 | 3 | 4 | 5 |
| NoiBene is useful to improve students' mental health | 1 | 2 | 3 | 4 | 5 |
| NoiBene is useful to know myself better | 1 | 2 | 3 | 4 | 5 |
| NoiBene is useful to improve myself | 1 | 2 | 3 | 4 | 5 |
| NoiBene is useful to improve awareness about mental health | 1 | 2 | 3 | 4 | 5 |
| Now please fill out the following open questions | | | | | |
| Which other topics would you like to find in NoiBene? | | | | | |
| Would you suggest NoiBene to other students? If not, why? | | | | | |
| Any feedback and suggestions | | | | | |

# Appendix-2. Feedback questionnaire (NB-WB condition)

| We ask you to answer the following questions concerning your experience with NoiBene. Please remember that the questionnaire is completely anonymous. Your opinion is essential to improve the program.  Please consider the following scale when answering the questions.   \| 1  Strongly disagree \| 2  Disagree \| 3  Neither agree or disagree \| 4  Agree \| 5  Strongly agree \| \| --- \| --- \| --- \| --- \| --- \| | | | | | |
| --- | --- | --- | --- | --- | --- | --- | --- | --- | --- | --- |
| NoiBene helped me to improve my well-being | 1 | 2 | 3 | 4 | 5 |
| NoiBene is useful to improve students' mental health | 1 | 2 | 3 | 4 | 5 |
| NoiBene is useful to know myself better | 1 | 2 | 3 | 4 | 5 |
| NoiBene is useful to improve myself | 1 | 2 | 3 | 4 | 5 |
| NoiBene is useful to improve awareness about mental health | 1 | 2 | 3 | 4 | 5 |
| Now please fill out the following open questions | | | | | |
| Which other topics would you like to find in NoiBene? | | | | | |
| Would you suggest NoiBene to other students? If not, why? | | | | | |
| Any feedback and suggestions | | | | | |
| We ask you to answer the following questions concerning your experience with the Tutor. Please remember that the questionnaire is completely anonymous. Your opinion is essential to improve the program.  Please consider the following scale when answering the questions.   \| 1  Strongly disagree \| 2  Disagree \| 3  Neither agree or disagree \| 4  Agree \| 5  Strongly agree \| \| --- \| --- \| --- \| --- \| --- \| | | | | | |
| I considered useful the presence of the Tutor | 1 | 2 | 3 | 4 | 5 |
| The Tutor helped me to understand misunderstanding contents | 1 | 2 | 3 | 4 | 5 |
| The Tutor helped me to better understand my feelings and thoughts | 1 | 2 | 3 | 4 | 5 |
| The Tutor helped me to carry on the program regularly | 1 | 2 | 3 | 4 | 5 |
| The Tutor helped me to do not lose my motivation | 1 | 2 | 3 | 4 | 5 |
| I would have preferred more control from my Tutor | 1 | 2 | 3 | 4 | 5 |
| Now please fill out the following open questions | | | | | |
| Would you have preferred another modality to be in touch with the Tutor (instead of the online video-call platforms)? If yes, which one? | | | | | |
| If you could choose, with which frequency would you like to have the appointments with the Tutor? | | | | | |
| Any feedback or opinions about the Tutor | | | | | |
| We ask you to answer the following questions concerning the specific modules you completed. Please remember that the questionnaire is completely anonymous. Your opinion is essential to improve the program.  Please consider the following scale when answering the questions.   \| 1  Strongly disagree \| 2  Disagree \| 3  Neither agree or disagree \| 4  Agree \| 5  Strongly agree \| \| --- \| --- \| --- \| --- \| --- \| | | | | | |
| Please consider the module “Commitment and Motivation” when answering the questions. | | | | | |
| The theoretical contents are understandable | 1 | 2 | 3 | 4 | 5 |
| The exercise was useful for helping to think and set my goals | 1 | 2 | 3 | 4 | 5 |
| I appreciate this module | 1 | 2 | 3 | 4 | 5 |
| I learnt from this module | 1 | 2 | 3 | 4 | 5 |
| Please consider the module “Self-awareness” when answering the questions. | | | | | |
| The theoretical contents were understandable | 1 | 2 | 3 | 4 | 5 |
| The quizzes were useful for helping me better understand the theoretical contents | 1 | 2 | 3 | 4 | 5 |
| The exercises were useful for helping me think about emotions and values | 1 | 2 | 3 | 4 | 5 |
| The self-monitoring was useful for helping me understand my feelings | 1 | 2 | 3 | 4 | 5 |
| I appreciate this module | 1 | 2 | 3 | 4 | 5 |
| I learnt from this module | 1 | 2 | 3 | 4 | 5 |
| Please consider the module “Psychological Bug” when answering the questions. | | | | | |
| The theoretical contents were understandable | 1 | 2 | 3 | 4 | 5 |
| The quizzes were useful for helping me better understand the theoretical contents | 1 | 2 | 3 | 4 | 5 |
| The exercises were useful for helping me think about vicious circles | 1 | 2 | 3 | 4 | 5 |
| I appreciate this module | 1 | 2 | 3 | 4 | 5 |
| I learnt from this module | 1 | 2 | 3 | 4 | 5 |
| Please consider the module “Perfectionism” when answering the questions. If you did not carry out this module, please move to the next section. | | | | | |
| The theoretical contents were understandable | 1 | 2 | 3 | 4 | 5 |
| The quizzes were useful for helping me better understand the theoretical contents | 1 | 2 | 3 | 4 | 5 |
| The exercises were useful for helping me think about my perfectionist vicious circles | 1 | 2 | 3 | 4 | 5 |
| The exercises were useful to foster a change in my thoughts and behaviours. | 1 | 2 | 3 | 4 | 5 |
| I appreciate this module | 1 | 2 | 3 | 4 | 5 |
| I learnt from this module | 1 | 2 | 3 | 4 | 5 |
| Please consider the module “Repetitive thinking” when answering the questions. If you did not carry out this module, please move to the next section. | | | | | |
| The theoretical contents were understandable | 1 | 2 | 3 | 4 | 5 |
| The quizzes were useful for helping me better understand the theoretical contents | 1 | 2 | 3 | 4 | 5 |
| The exercises were useful for helping me think about my repetitive thinking vicious circles | 1 | 2 | 3 | 4 | 5 |
| The self-monitoring tool was useful for helping me understand my repetitive thoughts | 1 | 2 | 3 | 4 | 5 |
| The exercises were useful to foster a change in my thoughts and behaviours. | 1 | 2 | 3 | 4 | 5 |
| I appreciate this module | 1 | 2 | 3 | 4 | 5 |
| I learnt from this module | 1 | 2 | 3 | 4 | 5 |
| Please consider the module “Avoidance” when answering the questions. If you did not carry out this module, please move to the next section. | | | | | |
| The theoretical contents were understandable | 1 | 2 | 3 | 4 | 5 |
| The quizzes were useful for helping me better understand the theoretical contents | 1 | 2 | 3 | 4 | 5 |
| The exercises were useful for helping me think about my avoidance vicious circles | 1 | 2 | 3 | 4 | 5 |
| The self-monitoring tool was useful for helping me understand my avoidance behaviours | 1 | 2 | 3 | 4 | 5 |
| The exercises were useful to foster a change in my thoughts and behaviours. | 1 | 2 | 3 | 4 | 5 |
| I appreciate this module | 1 | 2 | 3 | 4 | 5 |
| I learnt from this module | 1 | 2 | 3 | 4 | 5 |
| Please consider the module “Social skills” when answering the questions. | | | | | |
| The theoretical contents were understandable | 1 | 2 | 3 | 4 | 5 |
| The quizzes were useful for helping me better understand the theoretical contents | 1 | 2 | 3 | 4 | 5 |
| The self-monitoring tool was useful for helping me understand my communicative style | 1 | 2 | 3 | 4 | 5 |
| The exercises were useful to foster a change in my thoughts and behaviours. | 1 | 2 | 3 | 4 | 5 |
| I appreciate this module | 1 | 2 | 3 | 4 | 5 |
| I learnt from this module | 1 | 2 | 3 | 4 | 5 |
| Please consider the module “Self-realization” when answering the questions. | | | | | |
| The theoretical contents were understandable | 1 | 2 | 3 | 4 | 5 |
| The exercise was useful to set my goals | 1 | 2 | 3 | 4 | 5 |
| The study section gave me new insight to improve my study method | 1 | 2 | 3 | 4 | 5 |
| I appreciate this module | 1 | 2 | 3 | 4 | 5 |
| I learnt from this module | 1 | 2 | 3 | 4 | 5 |
| Please consider the module “Psychological First Aid” when answering the questions. If you did not carry out this module, please do not answer the following questions. | | | | | |
| The theoretical contents were understandable | 1 | 2 | 3 | 4 | 5 |
| The quizzes were useful for helping me better understand the theoretical contents | 1 | 2 | 3 | 4 | 5 |
| The contents and exercises gave me new insight to better understand myself | 1 | 2 | 3 | 4 | 5 |
| The exercises were useful to foster a change in my thoughts and behaviors. | 1 | 2 | 3 | 4 | 5 |
| I appreciate this module | 1 | 2 | 3 | 4 | 5 |
| I learnt from this module | 1 | 2 | 3 | 4 | 5 |
| Now please fill out the following open question | | | | | |
| Which section(s) of the module did you refer to? | | | | | |

# Table S-1. Protocol of NB-G intervention

| Meeting | Goal | Session organization |
| --- | --- | --- |
| 1st meeting | To introduce the program and to get to know each other; to improve awareness about wellbeing, emotions, need and values. | After an icebreaker game, the program and the specific aims of NoiBene are presented. Then, students have to set the goal in line with the intervention they desire to reach. Later, we introduce the concept of wellbeing according to the Ryff model (Ryff & Keyes, 1995) and Seligman model (Seligman, 2018). According to the explained model, they have to identify their hedonic and eudemonic pleasure activities. Next, after introducing emotion and need, a series of group activities are proposed to improve the ability to identify and distinguish emotion (in all its components) and need. Then, after the introduction of the role of cognitive appraisal in determining emotions (Dobson & Dobson, 2018) and the ABC technique (Ellis, 1995), a series of group activities are proposed to improve the ability to think about the link between emotion and personal thought and to discuss their feeling following the ABC model. Lastly, the concept of values (Hayes et al., 2006) is introduced. Some group and individual activities are proposed to identify their own values system and set actions in line with one's values. |
| 2nd meeting | To improve awareness about psychological vulnerability; to reduce repetitive thinking. | The meeting starts with a discussion about the learning techniques adopted in the past weeks. Then the concept of emotional, behavioral and cognitive control strategies (Dobson & Dobson, 2018) is explained. A group activity is proposed to identify one's own processes and attitudes that contribute to emotional suffering and relationship problems. After introducing rumination, worry, and self-criticism (Ehring & Watkins, 2008), a series of group activities are proposed to identify the negative consequences of repetitive thinking. Lastly, some individual exercises to stop overthinking are presented. |
| 3th meeting | To reduce perfectionism and avoidance. | The meeting starts with a discussion about the learning techniques adopted in the past weeks. After introducing behavioral and experiential avoidance (Chawla & Ostafin, 2007), some group activities are proposed to discuss the pros and cons of avoidance and focus on the negative consequences of avoidance. Then, the concept of safety strategies (Frank & Davidson, 2014) is introduced, followed by some group activity to discuss the pros and cons of safety strategies and some exercises to stop avoidance. Lastly, we introduce the concept of perfectionism (Egan et al., 2011). Group and individual activities to discuss the negative consequences of perfectionism, the pros and cons of perfectionism, and stop perfectionism are carried out. |
| 4th meeting | To improve social skills (active listening, assertive and empathic communication). | The meeting starts with a discussion about the learning techniques adopted in the past weeks. First, the concept of active listening (Rogers & Farson, 2021) is proposed, followed by some role-playings to improve active listening skill. Then, we introduce passive, aggressive, and assertive communication styles (Baggio, 2013). A series of group activities and role-playings are proposed to identify communication style consequences and to identify one's communication style. Lastly, the Nonviolent Communication Model (NVC - Rosenberg & Chopra, 2015) is presented, and some role-playings on the four steps of NVC are proposed. |
| 5th meeting | To improve social skills (active listening, assertive and empathic communication); to enhance positive social skills (constructive response, gratitude). | The meeting starts with a discussion about the learning techniques adopted in the past weeks. Then, some group activities and role-playings are proposed to improve assertiveness. Later, we discuss specific relational situations such as refusing requests and expressing and receiving criticism. Group activities are proposed to enhance such specific assertiveness skills. Then, we introduce the concept of gratitude and active and constructive response (Seligman et al., 2005). In the end, individual and group activities are proposed to improve gratitude and role-playing is proposed to learn how to respond actively and constructively. |
| 6th meeting | To provide information about healthy lifestyle; to increase the ability to plan and set goals; to improve study method. | The meeting starts with a discussion about the learning techniques adopted in the past weeks. In the beginning, psychoeducation about a healthy lifestyle (Olsen & Nesbitt, 2010) is proposed. Students are encouraging to link values and healthy lifestyle. Then, after introducing the SMART model to reach personal goals (Doran, 1981), a group activity is presented to set a goal following the SMART model, considering values (investigated in the first meeting) and a healthy lifestyle. In this context, a discussion about time management, procrastination and problem-solving processes is considered. In the end, different study methods and mnemonics are proposed; students are helped to identify their primary obstacles to the study process and, considering all the learning skills, they are encouraged to think about a problem-solving approach. |

# Table S-2. Protocol of NB-WB intervention

| Module | Aim | Theoretical contents |
| --- | --- | --- |
| Commitment and Motivation | Introduce the concept of well-being; to elicit commitment and motivation. | The module provides an introduction of the concept of wellbeing according to the Ryff model (Ryff & Keyes, 1995) and Seligman model (Seligman, 2018). Then students are asked to set the goal that they want to reach with the program; schedule a timetable of future access to the platform. |
| Self-awareness | To improve emotional awareness and to support basic individual needs and values. Especially, to help students become aware of their emotions and needs and to understand the personal thought and evaluation that underlie each emotion. | After an introduction about emotion and needs, some quizzes are proposed to implement the just learning contents. Then, after the introduction of the role of cognitive appraisal in determining emotions (Dobson & Dobson, 2018) and the ABC technique (Ellis, 1995) and a series of exercises are proposed to better understand the learning model. Then a self-monitoring tool is proposed: students are asked to use a personal diary, inspired by Ellis’ ABC technique (1995), designed to report one’s own emotive episodes. Lastly, there is an introduction about values (Hayes et al., 2006) and a series of exercises to discriminate values and goals and to identify one’s own values system. |
| Psychological Bugs | To identify student’s vulnerabilities. | The module starts with an introduction about emotional, behavioral and cognitive control strategies (Dobson & Dobson, 2018). Then some quizzes and exercises are proposed to identify one's own process and attitudes that contribute to emotional suffering and relationship problems. Lastly, students have to answer a series of questionnaires to identify the presence of the transdiagnostic factor that we take into account (i.e., perfectionism, worry, rumination, self-criticism and avoidance). Then students are directed towards the modules that are more suitable to their vulnerabilities. |
| Repetitive thinking | To help students to challenge repetitive thinking. | After an introduction about rumination, worry and self-criticism (Ehring & Watkins, 2008), students are asked to use a personal diary to monitor their repetitive thinking behavior. Then, some exercises to identify negative consequences of repetitive thinking are proposed. Lastly, students are encouraged to follow some techniques to challenge such thoughts. |
| Perfectionism | To help students to challenge perfectionism. | The module provides an introduction about perfectionism (Egan et al., 2011), followed by exercises to identify personal high standards and the positive and negative aspects for being a perfectionist. Then, a series of exercises are presented and students are encouraged to follow some techniques to challenge perfectionistic |
| Avoidance | To help students to challenge with avoidance. | After an introduction about behavioral and experiential avoidance (Chawla & Ostafin, 2007), a self-monitoring tool is proposed to monitor their avoidance behavior. Then, a series of exercises are proposed to identify negative consequences of avoidance and to stop avoidance; students are encouraged to follow some techniques to challenge such behaviour. |
| Social Skills | To promote the development of healthy and positive relationship through the improvement of some fundamental social competences: active listening, assertive communication, and gratitude. | The module provides an introduction about active listening (Rogers & Farson, 2021) and about passive, aggressive and assertive communication style (Baggio, 2013). Then an exercise to identify one's own communication style is proposed. After the presentation of the Nonviolent Communication Model (NVC - Rosenberg & Chopra, 2015) a series of self-monitoring tools are proposed to better understand the four steps of NVC. Then, are presented a series of exercises to help students to formulate assertive communication, to refuse requests assertively and to formulate criticism and reply to criticism in an assertive way. Lastly, a series of exercises about gratitudine are proposed. |
| Self-realization | To improve the goal setting and time management ability. To improve study method and to learn mnemonics. | The module starts with a theoretical introduction of the SMART model (Wade, 2009) to reach personal goals. Then students are asked to set a goal following the SMART model. Some in-depth pages about plan and procrastination are proposed. Then, different study methods and mnemonics are introduced, and students are helped to identify the best one that fits their needs and competences. |
| Psychological First Aid | To face with loneliness, rejection, failure, emotional loss.  To help students identify the first signs of clinically relevant conditions and to promote help-seeking behavior.  To provide information about healthy lifestyle. | This module contains a series of sections to cope with loneliness, rejection, failure, and emotional loss. The module also presents a series of relaxation and breath techniques. Also, it includes a section about anxiety and depression in terms of psychoeducation. Lastly, it includes psychoeducation about a healthy lifestyle (Olsen & Nesbitt, 2010). Students are encouraging to link values and goal setting with healthy lifestyle. |

# References

Baggio, F. (Ed.). (2013). *Assertività e training assertivo. Teoria e pratica per migliorare le capacità relazionali dei pazienti: Teoria e pratica per migliorare le capacità relazionali dei pazienti.* FrancoAngeli.

Chawla, N., & Ostafin, B. (2007). Experiential avoidance as a functional dimensional approach to psychopathology: An empirical review. *Journal of clinical psychology, 63*(9), 871-890. doi: 10.1002/jclp.20400

Doran, G. T. (1981). There’sa SMART way to write management’s goals and objectives. *Management review, 70*(11), 35-36.

Egan, S. J., Wade, T. D., & Shafran, R. (2011). Perfectionism as a transdiagnostic process: A clinical review. *Clinical psychology review, 31*(2), 203-212. doi: 10.1016/j.cpr.2010.04.009

Hayes, S. C., Luoma, J. B., Bond, F. W., Masuda, A., & Lillis, J. (2006). Acceptance and commitment therapy: Model, processes and outcomes. *Behaviour research and therapy, 44*(1), 1-25. doi: 10.1016/j.brat.2005.06.006

Rogers, C., & Farson, R. (2021). *Active listening.* Mockingbird Press LLC.
